# Excellent Microwave Absorption Properties Derived from the Synthesis of Hollow Fe_3_o_4_@Reduced Graphite Oxide (RGO) Nanocomposites

**DOI:** 10.3390/nano9020141

**Published:** 2019-01-22

**Authors:** Guangzhen Cui, Yanli Lu, Wei Zhou, Xuliang Lv, Jiangnan Hu, Guoyu Zhang, Guangxin Gu

**Affiliations:** 1 Key Laboratory of Science and Technology on Electromagnetic Environmental Effects and Electro-optical Engineering, The Army Engineering University of PLA, Nanjing 210007, China; cgzovezy@163.com (G.C.); xllu1957@126.com (X.L.); 2 The First Scientific Research Institute of WuXi, Wuxi 214035, China; wxz2397057180@163.com (Y.L.); liuhh1005@163.com (W.Z.); 3 Training Support Office, Troops 69006 of PLA, Xinjiang 835000, China; plasmx@126.com; 4 Teaching and Training Support Office, The Army Engineering University of PLA, Nanjing 210007, China; 5 Department of Materials Science, Fudan University, Shanghai 200433, China

**Keywords:** microwave absorption, solvothermal, heterojunction, hollow Fe_3_O_4_

## Abstract

Magnetic nanoparticles, such as Fe_3_O_4_ and Co_3_O_4_, play a vital role in the research on advanced microwave absorbing materials, even if problems such as high density and narrow band impedance matching are still unsolved. Herein, the study of lightweight hollow Fe_3_O_4_@reduced graphite oxide (RGO) nanocomposites synthesized via the solvothermal method is presented. The microstructure and crystal morphology of the materials were characterized by X-ray diffractometer (XRD), X-ray photoelectron spectroscopy (XPS), scanning electron microscopy (SEM), and transmission electron microscopy (TEM) analyses. Single crystalline hollow Fe_3_O_4_ spheres were grown onto RGO flakes, leading to the formation of heterojunction, which further influenced the microwave absorption properties. The latter were evaluated by standard microwave characterization in the frequency range of 2–18 GHz. It was found that, for a specific Fe_3_O_4_@0.125 g RGO composite, the minimum reflection loss can reach −41.89 dB at 6.7 GHz, while the reflection loss was less than −10 dB from 3.4 GHz to 13.6 GHz for a nanocomposite sample thickness in the range of 1–4 mm. The combination of these two materials thus proved to give remarkable microwave absorption properties, owing to enhanced magnetic losses and favorable impedance matching conditions.

## 1. Introduction

With the rapid advancement of science and technology, more and more electrical equipment and information systems have been used in various fields. However, such systems cause electromagnetic (EM) radiation, which is harmful to people’s health and affects the normal operation of electronic equipment. Therefore, the research on microwave absorbing materials (MAMs) has attracted extensive attention [1]. As an effective means to improve the survivability and penetration ability of weapon systems, the utilization of MAMs is extremely important for absorbing EM waves, reducing EM radiation, and improving the human living environment [2,3]. Nowadays, exploring new lightweight, reduced-thickness MAMs to meet the requirements of a wide absorption bandwidth, strong attenuation property with a good impedance match is very important [4,5,6].

In general, magnetite materials, including different types of ferrites [7], carbonyl iron [8], and inorganic magnetic microspheres [9], have been widely investigated and applied into practice for the merits of high-saturation magnetization and high coercivity, which contribute to magnetic energy loss [10]. Owing to Snoek’s limit at a high frequency, inverse spinel-type Fe_3_O_4_ with excellent magnetic properties hardly achieves the absorbing requirements in the gigahertz frequency range [11,12,13,14]. To enhance the microwave absorbing properties of Fe_3_O_4_ in the high frequency region, combining dielectric loss materials with Fe_3_O_4_ composite absorbers has been an effective way to improve the absorption properties of EM waves.

Recently, graphene [15], as a novel dielectric material, has attracted increasing attention owing to its low density and good physical and chemical properties [16]. Graphene oxide (GO) is a two-dimensional (2D) structure obtained by modifying the graphene sheet whose basal plane has hydroxyl groups and epoxide groups [17,18,19], which has a widespread application in chemistry [20], energy [21], catalysis [22], and environmental pollution control [23]. Nevertheless, GO materials that attenuate EM energy by means of dielectric loss are not favorable for EM absorption because of their poor impedance matching mechanism. It is well known that, based on the energy dissipation mechanism of the absorber, the microwave loss mechanism can be sorted into dielectric loss and magnetic loss, so effective complementarity between these factors is necessary to enhance microwave absorbing properties. Zong et al. [24] prepared a reduced graphene oxide-CoFe_2_O_4_ heterostructure composite synthesized by a facile route. The maximum absorption could reach −44.1 dB at 15.6 GHz and the absorption bandwidth with a reflection loss below −10 dB reached up to 4.7 GHz. Feng and co-workers prepared heterostructure Fe-Fe_3_O_4_@C core-shell composites, which displayed excellent microwave absorption via polymerization and calcination. The results showed that the optimal reflection loss can reach −29.3 dB at 12.6 GHz under a thickness of 3.9 mm [25]. Yang et al. [26] synthesized Fe_3_O_4_/Ppy/carbon nanotube (CNT) composites through the mingling of CNTs with Fe_3_O_4_/Ppy composites. The maximum absorption could reach −25.9 dB, and the effective bandwidth with a reflection loss less than −10 dB was about 4.5 GHz. 

Herein, we successfully prepared hollow Fe_3_O_4_@RGO nanocomposites by the solvothermal method, in order to obtain a composite with better EM absorption properties. The crystalline structure, morphology, microwave absorption properties, and absorption mechanism were systematically investigated. Furthermore, the electromagnetic impedance matching performance and the microwave attenuation ability were discussed.

## 2. Experimental

### 2.1. Synthesis of Hollow Fe_3_O_4_@RGO Nanocomposites

**Figure 1:**
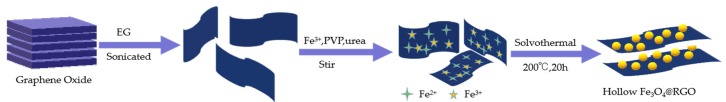
Schematic illustration of the fabrication of the hollow Fe_3_O_4_@reduced graphite oxide (RGO) nanocomposites.

A schematic illustration of the hollow Fe_3_O_4_@RGO composites is described in Figure 1. In this experiment, all chemicals were used as received, and the preparation process is demonstrated below [27,28]: First, 0.5 g of graphite oxide (GO) was dissolved in 80 mL of ethylene glycol (EG). After that, the solution was sonicated for 30 min, then 5.4 g of FeCl_3_·6H_2_O (Shanghai Aladdin Technology Co., Ltd., Shanghai, China) and 0.4 g of polyvinyl-pyrrolidone (PVP) were added. Then, after being stirred for 30 min, 2.5 g of urea (in a molar ratio Fe^3+^: urea = 1:2) was added. The obtained solution was transferred to a 100-mL Teflon-lined stainless-steel autoclave at 200 °C for 20 h. After that, the precipitates were washed with deionized water three times and dried at 60 °C for 12 h in a vacuum drying oven. As a comparison, 0.25 g of GO, 0.125 g of GO was dissolved in 80 mL of EG while maintaining other conditions, respectively.

### 2.2. Characterization and Measurement

An X-ray diffractometer (XRD, D8A Advance, BRUKER) was utilized to characterize the crystal structure of samples. X-ray photoelectron spectroscopy (XPS) data were obtained using a Thermo Fisher 0ESCALAB 250Xi. The morphology, size, and microstructure of the nanocomposites were examined on an FEIXL30 scanning electron microscope (SEM) and a G2F20 transmission electron microscope (TEM) [29]. The electromagnetic parameters were collected by a network analyzer (VNA, N5242A PNA-X, Agilent) through the classical coaxial measurement method by measuring in the frequency span from 10 MHz to 26.5 GHz. For the measurement of electromagnetic wave absorption properties, the samples were dispersed in paraffin homogeneously with a weight (wt%) ratio of 1:1 (sample/paraffin), and then the mixture was pressed into a cylindrical shape (*ϕ_out_* = 7 mm and *ϕ_in_* = 3 mm) with a thickness of only 3 mm. High requirements were needed for the test system to prepare samples, such as a smooth surface, flat without burrs or scratches, and no gaps between the inner and outer surfaces of the samples.

## 3. Results and Discussion

### 3.1. XRD and XPS Analyses

**Figure 2:**
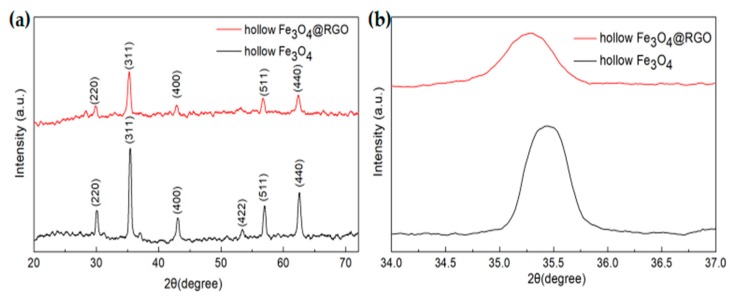
X-ray diffractometer (XRD) pattern of hollow Fe_3_O_4_@RGO (**a**) and the magnified image of the (311) peak (**b**).

For the sake of clarification, the XRD patterns of hollow Fe_3_O_4_ and hollow Fe_3_O_4_@RGO are illustrated in Figure 2. As could be observed, the typical peaks at 29.9°, 35.5°, 42.9°, 53.1°, 56.8°, and 62.5° could be readily indexed to the (220), (311), (400), (422), (511), and (440) planes of hollow Fe_3_O_4_; moreover, the face-centered cubic phase structure (JCPDS no. 89-2355) could also be seen [30]. Obviously, all peaks in the hollow Fe_3_O_4_@RGO curve are slightly weaker than those in the hollow Fe_3_O_4_ curve, which could be ascribed to the graphene content in the composites. In addition, the (311) peak of hollow Fe_3_O_4_@RGO shifted toward the lower angle of 35.3°, which is in contrast to that of the hollow Fe_3_O_4_ shown in the magnified picture of XRD patterns in Figure 2b. 

**Figure 3:**
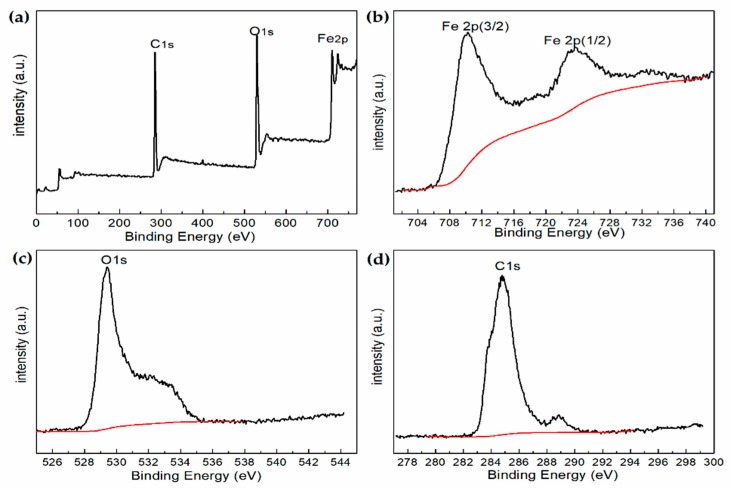
X-ray photoelectron spectroscopy (XPS) spectra of hollow Fe_3_O_4_@RGO (**a**), Fe2p spectrum (**b**), O1s spectrum (**c**), C1s spectrum (**d**).

To confirm the phases and structures of the hollow Fe_3_O_4_@RGO composites, XPS spectra were measured. Elements, including C, O, and Fe were found to be correlated with various atom contents, as illustrated by the large energy range spectrum (Figure 3a). In Figure 3b, the high-resolution spectrum of Fe could be deconvoluted into two peaks, related to Fe 2p3/2 and Fe 2p1/2, which correspond to the band energies of 710.9 eV and 724.3 eV, respectively, indicating that the mixing ratio of oxides of Fe (II) and Fe (III) is consistent with the reported value of Fe_3_O_4_ [31]. Figure 3c shows the O1s peaks at 529.1 eV and 532.1 eV, which could be attributed to the O element in Fe_3_O_4_ and RGO, respectively [32]. Moreover, the C1s peak (Figure 3d) at 285.1 eV could be attributed to the C element in RGO. Hence, the composites are suggested to be composed of Fe_3_O_4_ and RGO, revealing that hollow Fe_3_O_4_ was successfully grown on the RGO multilayer.

### 3.2. Morphology Analysis

**Figure 4:**
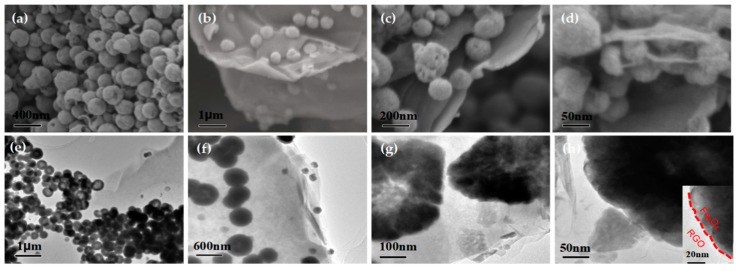
Scanning electron microscopy (SEM) image of hollow Fe_3_O_4_ (**a**); hollow Fe_3_O_4_@RGO (**b**–**d**); and transmission electron microscopy (TEM) image of hollow Fe_3_O_4_@RGO (**e**–**h**).

At the top of Figure 4, SEM and TEM images of the materials are shown, which contribute to the visualization of the morphology of both the hollow Fe_3_O_4_ and hollow Fe_3_O_4_@RGO nanocomposites. As presented in Figure 4a, all products have a hollow spherical structure with a uniform size, and the inner hollow structure can be observed in several broken pellets. Clearly, the cavity size is 130 nm, and the corresponding shell thickness is 35 nm, as can be seen in Figure 4d. Figure 4b–d provide representative images of the hollow Fe_3_O_4_@RGO, which suggest that the hollow Fe_3_O_4_ microspheres are adhered to the flaky RGO. Figure 4e–h display the overall structure of the hollow Fe_3_O_4_@RGO. Typically, the white area covered by gray or black areas is indicative of the hollowness of Fe_3_O_4_ microspheres, while the sample edge indicates the existence of RGO. In particular, it can be observed from Figure 4h that the RGO had coated the hollow Fe_3_O_4_. Notably, the Fe_3_O_4_ spheres in the surface of the hollow Fe_3_O_4_@RGO composites could improve the interaction between Fe_3_O_4_ and RGO. For the hollow Fe_3_O_4_@RGO composites, many charge carriers accumulated onto the interfaces, as shown in the inset of Figure 4h, which would lead to increased dielectric loss. Typically, the incident electromagnetic microwaves could be reflected and scattered between the multilayer interfaces, which would give rise to the dissipation of electromagnetic radiation energy [33].

### 3.3. Microwave Absorption Properties

**Figure 5:**
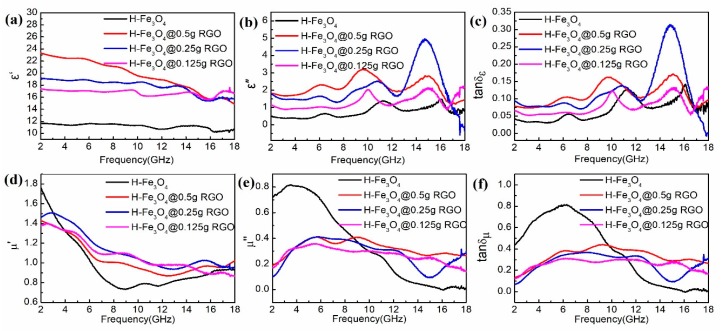
Real part of the relative complex permittivity (**a**); imaginary part of the relative complex permittivity (**b**); electric loss tangents (**c**); real part of the relative complex permeability (**d**); imaginary part of the relative complex permeability (**e**); and magnetic loss tangents of the prepared composites (**f**).

Generally, the electromagnetic microwave absorption mechanism is investigated by parameters such as the relative complex dielectric permittivity (*ε_r_* = *ε*’ – *jε*”), complex magnetic permeability (*μ_r_* = *μ*’ – *jμ*”), and corresponding tangents, as displayed in Figure 5. Among them, *ε*’ and *μ*’ are well known to represent the storage ability of electromagnetic energy, whereas *ε*” and *μ*” are linked with energy dissipation [34]. Furthermore, both dielectric loss and magnetic loss are responsible for energy attenuation in electromagnetic microwave absorption, which can be characterized by tanδ_ε_ and tanδ_μ_, respectively [35].

As can be observed from Figure 5a,b, the *ε*’ and *ε*” values of the hollow Fe_3_O_4_@RGO composites are higher than those of the hollow Fe_3_O_4_, owing to the addition of dielectric loss materials, which is particularly true for the hollow Fe_3_O_4_@0.5 g RGO composites. In detail, the *ε*’ values show a declining trend versus the changing frequency, whereas the *ε*” values exhibit a fluctuant variation trend, since the variation peaks appear at 6.5, 10, and 15 GHz across the whole range. Moreover, the permittivity curves indicate that the dielectric properties of the hollow Fe_3_O_4_ are greatly improved with the addition of RGO. Figure 5d,e show the *μ*’ and *μ*” values of the samples, among which the *μ*’ value presents a declining trend. Specifically, the *μ*’ value of the hollow Fe_3_O_4_ decreases dramatically in the range of 2–8 GHz, which is less than that of the hollow Fe_3_O_4_@RGO at the range of 4–18 GHz. Besides, the *μ*” values remain almost constant with the change in RGO concentration, except for the hollow Fe_3_O_4_. Figure 5c,f present the dielectric loss tangent and magnetic loss tangent of the samples, respectively. Notably, the dielectric loss tangent is less than 0.15, except for the hollow Fe_3_O_4_@0.25 g RGO in the range of 13–17 GHz, while the magnetic loss is mostly above 0.2. Obviously, the hollow Fe_3_O_4_@RGO composites possess a higher tanδ_μ_ at 2–18 GHz, which indicates that the magnetic loss contributes to the electromagnetic microwave absorption.

**Figure 6:**
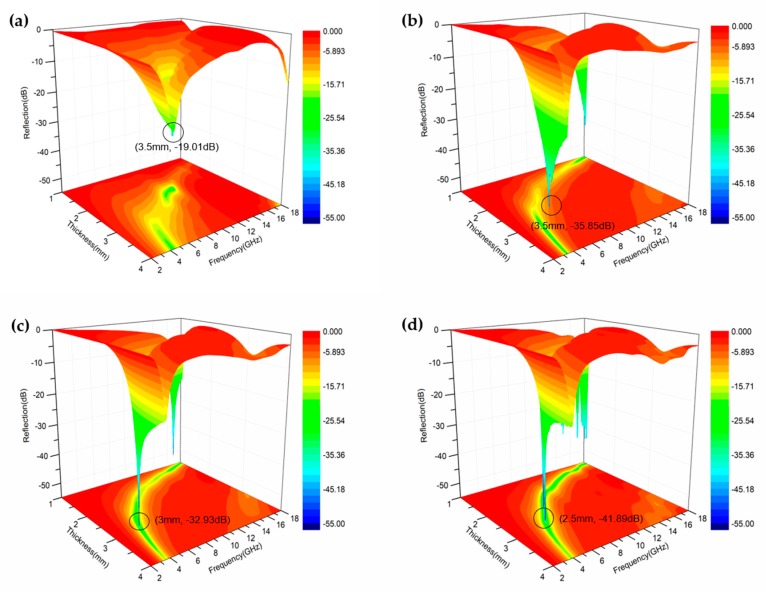
Three-dimensional (3D) presentations of reflection loss of the hollow Fe_3_O_4_ composite (**a**), hollow Fe_3_O_4_@0.5 g RGO composite (**b**), hollow Fe_3_O_4_@0.25 g RGO composite (**c**), and hollow Fe_3_O_4_@0.125 g RGO composite (**d**) at different thicknesses.

Based on the measured relative complex permeability and permittivity, the reflection loss (RL) of samples could be obtained according to the expressions listed below [36]:

(1) $$Z_{in}=Z_{0}\sqrt{\frac{\mu_{r}}{\varepsilon_{r}}}\tan h[j\frac{2\pi fd}{c}\sqrt{\mu_{r}\varepsilon_{r}}]$$

(2) $$R_{L}=20\log\left[\left|\frac{Z_{in}-Z_{0}}{Z_{in}+Z_{0}}\right|\right]$$

(3) $$Z_{0}=\sqrt{\frac{u_{0}}{\varepsilon_{0}}}$$

where *Z_in_*, *Z*_0_ are the normalized input and free space characteristic impedance, respectively; $\varepsilon_{0}$, *μ*_0_ are the permittivity and permeability of vacuum, respectively; *c* is the light velocity; *d* is the thickness of absorber; and *f* represents the microwave frequency. Figure 6a–d display the three-dimensional correlations among the RL, frequency, and thickness of the hollow Fe_3_O_4_, hollow Fe_3_O_4_@0.5 g RGO, hollow Fe_3_O_4_@0.25 g RGO, and hollow Fe_3_O_4_@0.125 g RGO composites. Notably, the minimum RL of the hollow Fe_3_O_4_ composites (Figure 6a) is only −19.01 dB, which indicates that the hollow Fe_3_O_4_ composites have a lower microwave absorption. In addition, it could be observed from Figure 6b that the minimum RL of the hollow Fe_3_O_4_@0.5 g RGO composites reached −35.85 dB at around 8.4 GHz, and the corresponding frequency bandwidth below −10 dB was 2.1 GHz (ranging from 7.4 GHz to 9.5 GHz). For the hollow Fe_3_O_4_@0.25 g RGO composites, the minimum RL was as high as −32.93 dB at 4.8 GHz at 3 mm, and the corresponding bandwidth of the RL below −10 dB was 1.8 GHz, as shown in Figure 6c. For the hollow Fe_3_O_4_@0.125 g RGO composites in Figure 6d, the minimum RL was −41.89 dB at 6.7 GHz at 2.5 mm. Moreover, the RL increased with the decrease in RGO, and the peak shifted to a higher frequency. It could be clearly observed when comparing the minimum RL of three samples that the hollow Fe_3_O_4_@0.125 g RGO composites displayed the optimal composite mode with a reduced thickness. More importantly, the top-level microwave absorption performances of the hollow Fe_3_O_4_@0.125 g RGO composites further underlined the strong magnetic loss of the material. All composites exhibited higher RLs compared with those of the single hollow Fe_3_O_4_, which proves that the combination of hollow Fe_3_O_4_ and RGO would induce a better performance of microwave absorption, thus demonstrating that RGO could greatly improve the absorption property owing to the dielectric loss.

**Figure 7:**
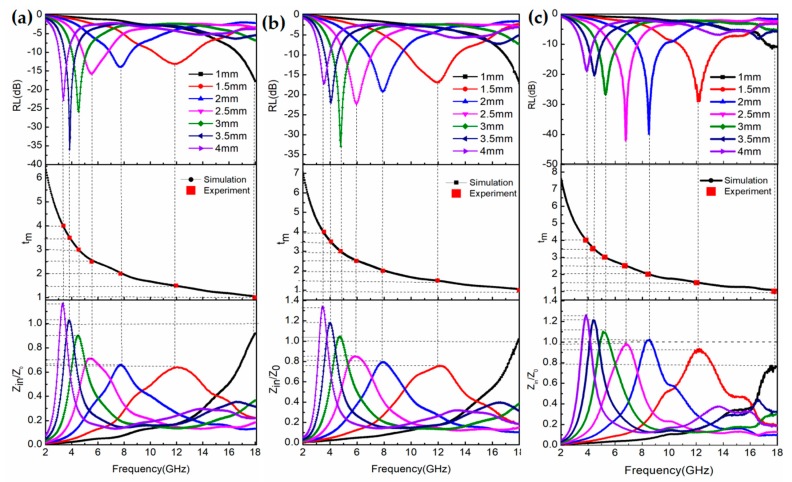
The reflection loss (RL) frequency curves, the relationship between the simulation thickness and the peak frequency, and relationship between *Z_in_*/*Z*_0_ and the electromagnetic (EM) wave frequency of the hollow Fe_3_O_4_@0.5 g RGO composites (**a**), hollow Fe_3_O_4_@0.25 g RGO composites (**b**), and hollow Fe_3_O_4_@0.125 g RGO composites (**c**).

Microwave absorption properties are greatly associated with thickness; typically, the RL of the hollow Fe_3_O_4_@RGO is calculated for an absorber thickness of 1–4 mm by Equations (1)–(3), as shown in Figure 7a–c. Remarkably, the bandwidths (RL below −10 dB) cover the range of 2.7–13.8 GHz for thicknesses ranging from 1 to 4 mm within the overall frequency for all the composites. Moreover, it can be noticed that for higher thicknesses the minimum RL shifts towards lower frequencies, while the absorption peaks of the hollow Fe_3_O_4_@RGO composites become sharper. The RL reached a maximum of −34.3 dB for the 3-mm thick hollow Fe_3_O_4_@0.25 g RGO composite. Noticeably, for lower thicknesses, higher losses occur for lower RGO contents within the composites, while an opposite trend can be established at higher thicknesses. A sort of ‘turning point’ can be thus conceived at a thickness of 3 mm (green curves in Figure 7) regarding the interaction between microwaves and this typology of RGO-reinforced composites. Specifically, the frequency was found to be partly related to the maximum RL at a certain thickness, as displayed on the quarter-wavelength cancellation model [37]. The matching equation is expressed below:

(4) $$t_{m}=\frac{n\lambda }{4}=nc/(4f_{m}\sqrt{\left|\mu_{r}\right|\left|\varepsilon_{r}\right|})$$

where *t_m_*, *λ*, and *f_m_* are the matching thickness of the absorber, the wavelength, and the absorption frequency of the electromagnetic microwave. The calculated matching thickness and the peak frequency are plotted below the RL curves, where the red squares represent the specific thickness in the range of 1–4 mm corresponding to the peak frequency. In addition, the simulation of *t_m_* vs. frequency for the hollow Fe_3_O_4_@RGO composites are also depicted based on the above equation. The red squares appear on the simulation curve, suggesting that the absorber thickness could be tailored to design the microwave absorption materials.

Furthermore, better impedance matching maximizes the penetration of electromagnetic waves into the material and reduces the direct reflection on the surface of the material [38]. When the value of the impedance matching characteristics (*Z*) is equal or close to 1, zero reflection is achieved, which is expressed as the following equation:

(5) $$Z_{in}=\sqrt{\left|\frac{\mu_{r}}{\varepsilon_{r}}\right|}\tan h\left[\left(j\frac{2\pi fd}{c}\right)\sqrt{\mu_{r}\varepsilon_{r}}\right]$$

The *Z* value of hollow Fe_3_O_4_@0.125 g RGO composites (Figure 7c) is almost equal to 1. Figure 7 reveals the amount of electromagnetic waves entering into the absorber and the good impedance matching in comparison with hollow Fe_3_O_4_@0.5 g RGO and hollow Fe_3_O_4_@0.25 g RGO composites.

Furthermore, magnetic loss is also closely correlated with natural resonance, eddy current resonance, hysteresis loss, and domain wall displacement. Generally, the eddy current coefficient (*C*_0_) is almost a constant in accordance with the following equation when only magnetic loss is caused by the eddy current at the frequency range of 2–18 GHz.

(6) $$C_{0}=\mu^{\prime \prime }{\left(\mu^{\prime }\right)}^{-2}f^{-1}$$

**Figure 8:**
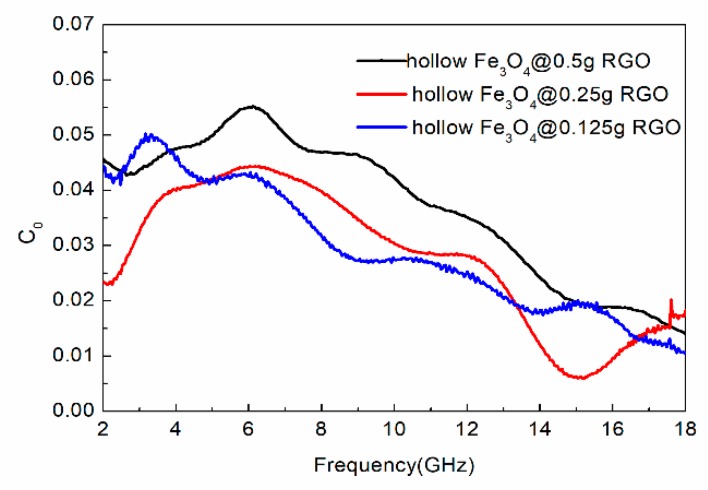
The *C*_0_-*f* curve of the hollow Fe_3_O_4_@RGO composite.

As shown in Figure 8, the *C_0_-f* curve presents an obviously declining trend at the frequency range of 2–18 GHz, which means that the attenuation of electromagnetic microwaves is not caused by eddy current resonance. However, the hysteresis loss is always inoperative in the weak field, while the domain wall resonance loss commonly appears in the MHz frequency range [39]. Hence, a conclusion could be drawn that the magnetic loss is mainly produced by natural resonance.

Table 1 lists some reported microwave absorption composites of the representative Fe_3_O_4_ material-based, graphene material-based, and Fe_3_O_4_@0.125 g RGO composite prepared in this work. Notably, the hollow Fe_3_O_4_@0.125 g RGO composites not only had a wide effective absorption bandwidth, but also displayed a promising negative RL value owing to this special hollow structure. Furthermore, the as-fabricated hollow Fe_3_O_4_@RGO properties could enhance the absorption performance and suit the requirements of ideal MAMs.

The microwave absorption performance of the hollow Fe_3_O_4_@RGO could be ascribed to the following reasons. (a) The outstanding magnetic hollow Fe_3_O_4_ sphere would induce a magnetic loss. (b) The main loss mechanism derives from dielectric loss rather than magnetic loss. Normally, dielectric loss is related to electronic dipole polarization and interfacial polarization. Firstly, electron migration, such as Fe^2+^ and Fe^3+^ ions, would induce dipole polarization in the composites. Secondly, the different neighboring phases, including dielectric constant and conductivity, have a significant effect on interfacial polarization. For the hollow Fe_3_O_4_@RGO composites, many charge carriers accumulated onto the interfaces, as shown in the TEM images, which aggravated the dielectric loss. (c) The interaction of dielectric loss and magnetic loss is also an important factor for the improvement of the microwave absorption performance; as a result, the RGO nanosheets modified by magnetic hollow Fe_3_O_4_ spheres have excellent absorption abilities.

## 4. Conclusions

In summary, hollow Fe_3_O_4_@RGO composites were successfully synthesized by the one-step solvothermal reaction route, which exhibited excellent microwave absorption properties in terms of the maximum RL value and the absorption bandwidth in the range of 2–18 GHz through tuning the hollow [Fe_3_O_4_]/[RGO] ratio. The surfaces of RGO were densely covered with hollow Fe_3_O_4_ spheres ~130 nm in diameter, with a corresponding shell thickness of ~35 nm, as shown by the SEM and TEM images. Specifically, the maximum RL could reach −41.89 dB at 6.7 GHz and the absorption bandwidth below −10 dB was as wide as 4.2 GHz at a thickness of 2.5 mm. The excellent performance of the novel composites could be ascribed to the strong magnetic loss and favorable impedance matching, and such microwave absorbers with strong absorption and a wide frequency band have shown great application potential in military and commercial fields.

## Figures and Tables

**Table 1:** Microwave absorption performances of various Fe_3_O_4_ nanoparticles in previous reports compared with hollow Fe_3_O_4_@RGO composites.

Sample	RL (dB)	Effective Bandwidth (GHz) (RL < 10 dB)	Thickness (mm)	Wt (%)	Reference
Fe_3_O_4_/5 wt% CNTs	−51.3	3.9	4.4	50	[40]
RGO/Fe_3_O_4_/ZnO	−57.0	5	2	33	[41]
CNTs/Fe_3_O_4_/rGO/C	−54.43	3.2	1.9	20	[42]
graphene@Fe_3_O_4_@PANI@TiO_2_	−41.8	3.5	1.6	50	[43]
graphene@Fe_3_O_4_@WO_3_@PANI	−46.7	1.8	1.5	30	[44]
Fe_3_O_4_/SiO_2_/graphene	−27.1	2.5	1.5	50	[45]
RGO/CoFe_2_O_4_/MWCNT	−46.8	3.4	1.6	50	[46]
PANI/Fe_3_O_4/_RGO	−36.5	5	2.5	40	[47]
hollow Fe_3_O_4_@RGO	−41.89	4.2	2.5	50	This work

## References

[1] Yu S., Hong V.M., Wang F., Xiao Z., Li C., Kong L., Que W., Zhou K. (2018). Synthesis and application of iron-based nanomaterials as anodes of lithium-ion batteries and supercapacitors. J. Mater. Chem. A.

[2] Wang L., Huang Y., Li C., Chen J., Sun X. (2015). Hierarchical composites of polyaniline nanorod arrays covalently-grafted on the surfaces of graphene@Fe_3_O_4_@C with high microwave absorption performance. Compos. Sci. Technol..

[3] Luo J., Xu Y., Yao W., Jiang C., Xu J. (2015). Synthesis and microwave absorption properties of reduced graphene oxide-magnetic porous nanospheres-polyaniline composites. Compos. Sci. Technol..

[4] Kong L., Yin X., Yuan X., Zhang Y., Liu X., Cheng L., Zhang L. (2014). Electromagnetic wave absorption properties of graphene modified with carbon nanotube/poly(dimethyl siloxane) composites. Carbon.

[5] Chen S., Chi M., Zhu Y., Gao M., Wang C., Lu X. (2018). A Facile synthesis of superparamagnetic Fe_3_O_4_ nanofibers with superior peroxidase-like catalytic activity for sensitive colorimetric detection of L-cysteine. Appl. Surf. Sci..

[6] Feng J., Zong Y., Sun Y., Zhang Y., Yang X., Long G., Wang Y., Li X., Zheng X. (2018). Optimization of porous FeNi_3_/N-GN composites with superior microwave absorption performance. Chem. Eng. J..

[7] Zhang J., Ma J., Fan X., Peng W., Zhang G., Zhang F., Li L. (2017). Graphene supported Au-Pd-Fe_3_O_4_ alloy trimetallic nanoparticles with peroxidase-like activities as mimic enzyme. Catal. Commun..

[8] Zhang H.B., Yan Q., Zheng W.G., He Z., Yu Z.Z. (2011). Tough graphene-polymer microcellular foams for electromagnetic interference shielding. ACS Appl. Mater. Interfaces.

[9] Dong N., He F., Xin J., Wang Q., Lei Z., Su B. (2015). Preparation of CoFe_2_O_4_ magnetic fiber nanomaterial via a template-assisted solvothermal method. Mater. Lett..

[10] Wang S., Zhao Y., Xue H., Xie J., Feng C., Li H. (2018). Preparation of flower-like CoFe_2_O_4_@graphene composites and their microwave absorbing properties. Mater. Lett..

[11] Qu B., Zhu C., Li C., Zhang X., Chen Y. (2016). Coupling Hollow Fe_3_O_4_–Fe Nanoparticles with Graphene Sheets for High-Performance Electromagnetic Wave Absorbing Material. ACS Appl. Mater. Interfaces.

[12] Wang Y., Wu X., Zhang W., Luo C., Li J., Wang Q., Wang Q. (2018). Synthesis of polyaniline nanorods and Fe_3_O_4_ microspheres on graphene nanosheets and enhanced microwave absorption performances. Mater. Chem. Phys..

[13] Sarkar D., Ghosh A., Rakshit R., Mandal K. (2015). Magnetic properties of Fe_3_O_4_ nano-hollow spheres. J. Magn. Magn. Mater..

[14] Torkian S., Ghasemi A., Shoja Razavi R. (2017). Cation distribution and magnetic analysis of wideband microwave absorptive CoxNi_1_−xFe_2_O_4_ ferrites. Ceram. Int..

[15] Li S., Wu Q., Ma P., Zhang Y., Song D., Wang X., Sun Y. (2018). A sensitive SPR biosensor based on hollow gold nanospheres and improved sandwich assay with PDA-Ag@Fe_3_O_4_/rGO. Talanta.

[16] Liang Y., He X., Chen L., Zhang Y. (2014). Facile preparation of graphene/Fe_3_O_4_/TiO_2_ multifunctional composite for highly selective and sensitive enrichment of phosphopeptides. RSC Adv..

[17] Micheli D., Pastore R., Delfini A., Giusti A., Vricella A., Santoni F., Marchetti M., Tolochko O., Vasilyeva E. (2017). Electromagnetic characterization of advanced nanostructured materials and multilayer design optimization for metrological and low radar observability applications. Acta Astro.

[18] Micheli D., Pastore R., Vricella A., Marchetti M. (2017). Matter’s Electromagnetic Signature Reproduction by Graded-Dielectric Multilayer Assembly. IEEE Trans. Microw. Theory Technol..

[19] Mazzoli A., Corinaldesi V., Donnini J., Perna C., Micheli D., Vricella A., Pastore R., Bastianelli L., Moglie F., Primiani V.M. (2018). Effect of graphene oxide and metallic fibers on the electromagnetic shielding effect of engineered cementitious composites. J. Build. Eng..

[20] Yazici E., Yanik S., Yilmaz M.B. (2017). Graphene oxide nano-domain formation via wet chemical oxidation of graphene. Carbon.

[21] Lv K., Zhao G., Wang X. (2012). A brief review of graphene-based material synthesis and its application in environmental pollution management. Chin. Sci. Bull..

[22] Zhang Y., Qi F., Li Y., Zhou X., Sun H., Zhang W., Liu D., Song X.M. (2017). Graphene oxide quantum dot-sensitized porous titanium dioxide microsphere: Visible-light-driven photocatalyst based on energy band engineering. J. Colloid Interface Sci..

[23] Pei S., Wei Q., Huang K., Cheng H.M., Ren W. (2018). Green synthesis of graphene oxide by seconds timescale water electrolytic oxidation. Nat. Commun..

[24] Zong M., Huang Y., Zhang N. (2015). Reduced graphene oxide-CoFe_2_O_4_ composite: Synthesis and electromagnetic absorption properties. Appl. Surf. Sci..

[25] Feng A., Jia Z., Zhao Y., Lv H. (2018). Development of Fe/Fe_3_O_4_@C composite with excellent electromagnetic absorption performance. J. Alloys Compd..

[26] Yang R.B., Reddy P.M., Chang C.J., Chen P.A., Chen J.K., Chang C.C. (2016). Synthesis and characterization of Fe_3_O_4_/polypyrrole/carbon nanotube composites with tunable microwave absorption properties: Role of carbon nanotube and polypyrrole content. Chem. Eng. J..

[27] Li W., Qiao X., Zheng Q., Zhang T. (2011). One-step synthesis of MFe_2_O_4_ (M=Fe, Co) hollow spheres by template-free solvothermal method. J. Alloys Compd..

[28] Sui M., Sun X., Lou H., Li X., Lv X., Li L., Gu G. (2018). Synthesis of hollow Fe_3_O_4_ particles via one-step solvothermal approach for microwave absorption materials: Effect of reactant concentration, reaction temperature and reaction time. J. Mater. Sci. Mater. Electron..

[29] Wang Y., Gao X., Wu X., Zhang W., Wang Q., Luo C. (2018). Hierarchical ZnFe_2_O_4_@RGO@CuS composite: Strong absorption and wide-frequency absorption properties. Ceram. Int..

[30] Zhou X., Zhao G., Liu Y. (2014). Uniform hollow magnetite spheres: Facile synthesis, growth mechanism, and their magnetic properties. Mater. Res. Bull..

[31] Lin C.-R., Chen I.H., Wang C.-C., Chen M.-L. (2011). Synthesis and characterization of magnetic hollow nanocomposite spheres. Acta. Mater..

[32] Liu X., Ma Y., Zhang Q., Zheng Z., Wang L.S., Peng D.L. (2018). Facile synthesis of Fe_3_O_4_/C composites for broadband microwave absorption properties. Appl. Surf. Sci..

[33] Shu R., Zhang G., Zhang J., Wang X., Wang M., Gan Y., Shi J., He J. (2018). Synthesis and high-performance microwave absorption of reduced graphene oxide/zinc ferrite hybrid nanocomposite. Mater. Lett..

[34] Zhan Y., Wang J., Zhang K., Li Y., Meng Y., Yan N., Wei W., Peng F., Xia H. (2018). Fabrication of a flexible electromagnetic interference shielding Fe_3_O_4_@reduced graphene oxide/natural rubber composite with segregated network. Chem. Eng. J..

[35] Huang X., Zhang J., Rao W., Sang T., Song B., Wong C. (2016). Tunable electromagnetic properties and enhanced microwave absorption ability of flaky graphite/cobalt zinc ferrite composites. J. Alloys Compd..

[36] Zhang X., Zhu W., Zhang W., Zheng S., Qi S. (2018). Preparation of TiO_2_/Fe_3_O_4_/CF composites for enhanced microwave absorbing performance. J. Mater. Sci. Mater. Electron..

[37] Kong L., Wang C., Yin X., Fan X., Wang W., Huang J. (2017). Electromagnetic wave absorption properties of a carbon nanotube modified by a tetrapyridinoporphyrazine interface layer. J. Mater. Chem. C.

[38] Lv H., Liang X., Cheng Y., Ji G., Tang D., Zhang B., Zhang H., Du Y. (2015). Facile synthesis of porous coin-like iron and its excellent electromagnetic absorption performance. RSC Adv..

[39] Liu Q., Liu X., Feng H., Shui H., Yu R. (2017). Metal organic framework-derived Fe/carbon porous composite with low Fe content for lightweight and highly efficient electromagnetic wave absorber. Chem. Eng. J..

[40] Zhu L., Zeng X., Chen M., Yu R. (2017). Controllable permittivity in 3D Fe_3_O_4_/CNTs network for remarkable microwave absorption performances. RSC Adv..

[41] Zhang N., Huang Y., Wang M. (2018). 3D ferromagnetic graphene nanocomposites with ZnO nanorods and Fe_3_O_4_ nanoparticles co-decorated for efficient electromagnetic wave absorption. Compos. B Eng..

[42] Zhang K., Zhang Q., Gao X., Chen X., Wang Y., Li W., Wu J. (2018). Effect of absorbers’ composition on the microwave absorbing performance of hollow Fe_3_O_4_ nanoparticles decorated CNTs/graphene/C composites. J. Alloys Compd..

[43] Liu P., Huang Y., Yang Y., Yan J., Zhang X. (2016). Sandwich structures of graphene@Fe_3_O_4_@PANI decorated with TiO_2_ nanosheets for enhanced electromagnetic wave absorption properties. J. Alloys Compd..

[44] Wang Y., Wu X., Zhang W., Luo C., Li J., Wang Q. (2017). 3D heterostructure of graphene@Fe_3_O_4_@WO_3_ @PANI: Preparation and excellent microwave absorption performance. Synth. Meter..

[45] Liu X., Chen Y., Hao C., Ye J., Yu R., Huang D. (2016). Graphene-enhanced microwave absorption properties of Fe_3_O_4_/SiO_2_ nanorods. Compos. A Appl. Sci. Manuf..

[46] Zhang K., Gao X., Zhang Q., Li T., Chen H., Chen X. (2017). Preparation and microwave absorption properties of asphalt carbon coated reduced graphene oxide/magnetic CoFe_2_O_4_ hollow particles modified multi-wall carbon nanotube composites. J. Alloys Compd..

[47] Chen T., Qiu J., Zhu K., Che Y., Zhang Y., Zhang J., Li H., Wang F., Wang Z. (2014). Enhanced electromagnetic wave absorption properties of polyaniline-coated Fe_3_O_4_/reduced graphene oxide nanocomposites. J. Mater. Sci. Mater. Electron..

